# Supplementary material for: High-performance and scalable on-chip digital Fourier transform spectroscopy
Source: Nat Commun. 2018 Oct 23;9:4405. doi: 10.1038/s41467-018-06773-2 (PMC6199339; doi:10.1038/s41467-018-06773-2)
Supplement: Supplementary file 2 — Supplementary Information [file 41467_2018_6773_MOESM2_ESM.pdf]

High-performance and scalable on-chip digital Fourier transform spectroscopy

Kita *et al.*

Supplementary Information

Supplementary Note 1: Elastic-D1 Spectral Reconstruction Method

Given the measured interferogram $\mathbf{y}$ (of size $N \times 1$) and a calibration matrix $\mathbf{A}$ of size $N \times D$, we seek to accurately reconstruct the input optical signal $\boldsymbol{x}$ that obeys:

$$\mathbf{y} = \mathbf{Ax} \qquad (1)$$

where $D \gg N$, and in our case $D = 801$, $N = 64$. For our 64-channel device, there are two types of signals available to us for testing the quality of optical reconstruction: (1) laser lines that are characterized by sparse spectra, and (2) broadband sources (like spontaneous emission from an EDFA) with a broad spectrum (non-sparse). Since the problem we are solving is underconstrained, there are infinite solutions $\mathbf{x}$ that solve Supplementary Equation 1. However, we can place constraints on the sparsity and magnitude of the spectrum and prevent over-fitting issues by minimizing the L_1 and L_2 norms of $\mathbf{x}$[1]:

$$\min_{\mathbf{x}} \{ ||\mathbf{y} - \mathbf{Ax}||^2 + \alpha_1 ||\mathbf{x}||_1 + \alpha_2 ||\mathbf{x}||_2^2 \} \qquad (2)$$

where α_1 and α_2 are the corresponding hyperparameters. For an arbitrary optical input, we find that the "smoothness" of the spectrum is an important characteristic of the spectra and a good regularizer. To induce the appropriate amount of smoothness, characterized by the first-derivative of the spectrum, we used the finite difference matrix $\mathbf{D}$ to define the following regularizer $||\mathbf{Dx}||_2^2$. We cast our reconstruction problem with L_1 norm, L_2 norm, and the first-derivative smoothness prior as follows:

$$\min_{\mathbf{x},\mathbf{x}>0} \{ ||\mathbf{y} - \mathbf{Ax}||^2 + \alpha_1 ||\mathbf{x}||_1 + \alpha_2 ||\mathbf{x}||_2^2 + \alpha_3 ||\mathbf{Dx}||_2^2 \} \qquad (3)$$

Using $||\mathbf{Mx}||_2^2 = \mathbf{x}^{\boldsymbol{T}}\mathbf{M}^{\boldsymbol{T}}\mathbf{Mx}$, and the fact that our spectrum is non-negative (and thus $||\mathbf{x}||_1 = \mathbf{1}^T\mathbf{x}$), we may rewrite Supplementary Equation 3 as a non-negative quadratic program:

$$\min_{\mathbf{x},\mathbf{x}>0} \{ \mathbf{x}^T(\mathbf{A}^T\mathbf{A} + \alpha_2\mathbf{I} + \alpha_3\mathbf{D}^T\mathbf{D})x + (\alpha_2\mathbf{1} - 2\mathbf{A}^T\mathbf{y})^T\mathbf{x} \} \qquad (4)$$

The above form is easily computed with standard quadratic program solvers[2]. With this method of solving for the signal $\boldsymbol{x}$, the last step is to determine the suitable hyperparameters α_1, α_2, and α_3 that correspond to the correct input. However, since we don't have access to the true spectrum, we use a standard holdout cross-validation technique, which requires only two successive measurements of the interferogram, characterized by the same input signal with different noise. The cross-validation technique is as follows: with two independent measurements $\mathbf{y_1}$ and $\mathbf{y_2}$ of the same source, and given two measurements of the basis $\mathbf{A_1}$ and $\mathbf{A_2}$ (performed only once in advance as a calibration step for the spectrometer), we solve for $\mathbf{x_1}$ via Supplementary Equation 4 for a suitably large range of hyperparameter values, and arguments $\mathbf{y_1}$ and $\mathbf{A_1}$. We then choose the spectrum $\mathbf{x_1}$ corresponding to the unique set of α's that maximize the coefficient of determination R^2 (see Supplementary Equation 5 below) between the second measurement $\mathbf{y_2}$ and the value $\mathbf{A_2x_1}$:

$$\max_{\alpha_{1,2,3}} \{ R^2(\mathbf{y_2},\ \mathbf{A_2x_1}) \} = \max_{\alpha_{1,2,3}} \{ 1 - \frac{\sum_{i=0}^{n-1}\left(y_{2,i} - (\mathbf{A_2x_1})_i\right)^2}{\sum_{i=0}^{n-1}\left(y_{2,i} - \langle \mathbf{y_2} \rangle\right)^2} \} \qquad (5)$$

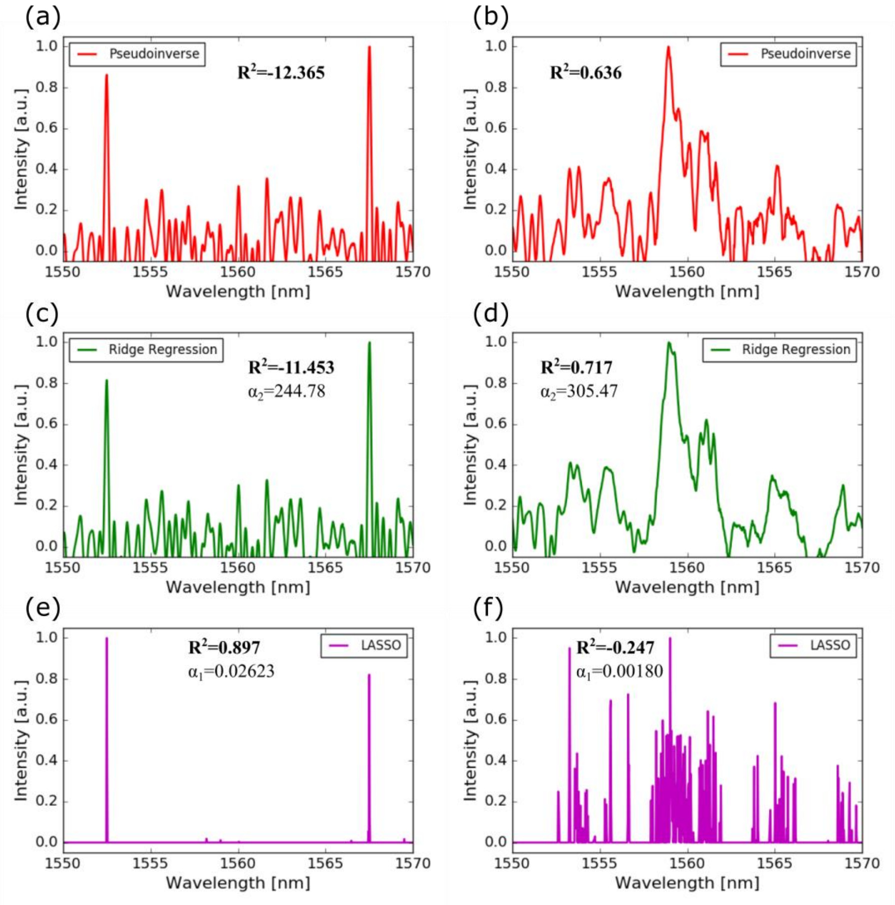

Supplementary Figure 1. Comparison of reconstruction techniques. Images of reconstructed spectra for two different optical inputs: two CW laser lines with 15 nm wavelength detuning (a,c,e) (as depicted in Figure 3 bottom) and a broadband signal generated by EDFA amplified spontaneous emission (b,d,f) (Figure 4c). The reconstruction techniques shown are: pseudoinverse (a,b), ridge regression (c,d), and LASSO (e,f). The R^2 scores and associated hyperparameters (which were selected through cross-validation) are shown as insets.

where in the above the subscript i specifies the specific interferogram measurement (out of $n = 64$ total measurements) and angled brackets denote the average value ($\langle \mathbf{y_2} \rangle = (1/n)\sum_{i=0}^{n-1} y_i$). The coefficient of determination value of 1 corresponds to a perfect match between inputs, and a value less than 0 means the measurement ($\mathbf{y_2}$) is more accurately fit by a straight line than it is fit by the model ($\mathbf{A_2 x_1}$). Maximizing the coefficient of determination above guarantees that our computed spectrum $\mathbf{x_1}$ corresponds to a back-computed interferogram that closely matches a second experimental measurement of the interferogram. This technique allows for the unique determination of a set of hyperparameters that minimize the impact of experimental noise on the spectral reconstruction process. In addition, the hyperparameter search method is trivially parallelizable to systems with multiple processors.

The reader should note that any regularizer can be used with elastic-D_1 if one can encode this new regularizer with a matrix $\mathbf{R}$ and substitute $\mathbf{D_1}$ with $\mathbf{R}$. Thus, our method suggests that any

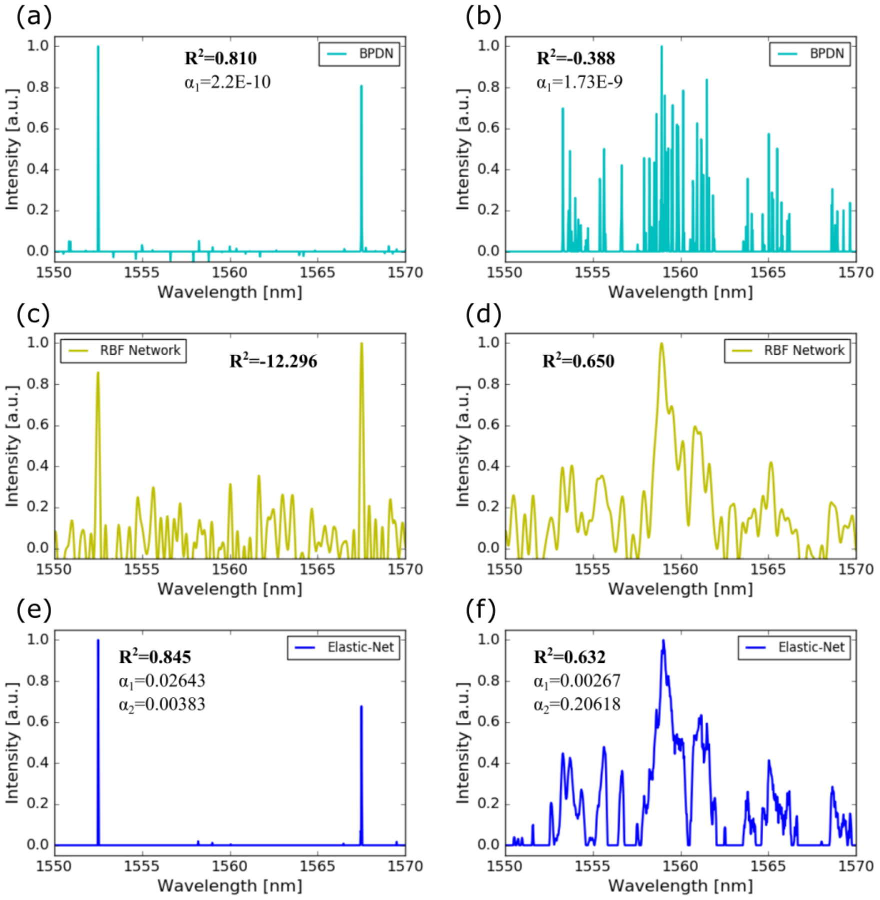

Supplementary Figure 2. Comparison of reconstruction techniques. Images of reconstructed spectra for two different optical inputs: two CW laser lines with 15 nm wavelength detuning (a,c,e) (as depicted in Figure 3 bottom) and a broadband signal generated by EDFA amplified spontaneous emission (b,d,f) (Figure 4c). The reconstruction techniques shown are: BPDN (a,b), RBF Network (c,d), and elastic net (e,f). The R^2 scores and associated hyperparameters (which were selected through cross-validation) are shown as insets.

regularizer $\mathbf{R}$ can be used to encode any combination of new priors that can be expressed as a matrix. In addition we want to point out the trivial extension to include additional regularizer terms $\mathbf{x}^T\mathbf{R}_{\mathrm{i}}^T\mathbf{R}_{\mathrm{i}}\mathbf{x}$ to elastic-D_1. This can be easily done by defining the optimization problem

$$\left|\left|\mathbf{Ax}-\mathbf{y}\right|\right|_2^2+\alpha_1\left|\left|\mathbf{x}\right|\right|_1+\alpha_2\left|\left|\mathbf{x}\right|\right|_2^2+\beta_1\mathbf{x}^T\mathbf{R}_{\mathrm{i}}^T\mathbf{R}_{\mathbf{1}}\mathbf{x}+\cdots+\beta_k\mathbf{x}^T\mathbf{R}_k^T\mathbf{R}_{\boldsymbol{k}}\mathbf{x} \qquad (6)$$

with β_i denoting the i-th hyperparameter for each of the k regularizers encoded by the matrix $\mathbf{R}_{\boldsymbol{i}}$. This can be used to include additional regularizers, and can be solved using hold-out cross validation and with the convex libraries used before. As long as $k = O(1)$, the algorithm will remain polynomial time. In practice, we predict k should be at most 2 or 3. If one adds many more regularizers the algorithm runtime becomes exponential in k. To avoid this one would have to use techniques different from hold-out cross validation.

Supplementary Note 2: Insertion loss measurements of the dFT spectrometers

In order to estimate the total loss of the 64-channel dFT spectrometer, as well as the switching loss per stage, we measured the optical power before and after a set of reference waveguides, as well as dFT structures with 1, 2, and 3 stages (corresponding to 4-channel, 16-channel, and 64-channel dFT spectrometers), as illustrated in Supplementary Figure 3.

We determined the polarization-dependent insertion loss of the photonic components as a function of wavelength by scanning a single frequency tunable laser. We first performed a reference measurement of the grating coupler and fiber losses using a loopback waveguide on the chip (two grating couplers connected only by a straight waveguide). The fiber-to-fiber loopback loss was measured at 6.7 ± 0.7 dB across the 1550 – 1570 nm wavelength range. Next, we measured the insertion loss across the 64 channel dFT spectrometer by sending light into port “64ch_in” and measuring the outputs at both top and bottom tap-ports (labelled “64ch_top” and “64ch_bot” in Supplementary Figure 3c. When the spectrometer is powered on, the top and bottom tap-ports have a relatively flat spectral response (in contrast to the “64ch_out” port, which exhibits the distinct interference fringes). Since the light in the final 2 × 2 MMI is split 50:50 amongst the tap port and the final 2 × 1 combiner, the total optical power that would exit the 2 × 1 combiner is equivalent to the sum of the two tap-ports (neglecting the loss of the 2 × 1 MMI).

Summing the two tap-ports and normalizing by the reference waveguide losses, the measured insertion loss across the spectrum was 9.1 ± 1.7 dB. The same chip contained 1- and 2-stage dFT

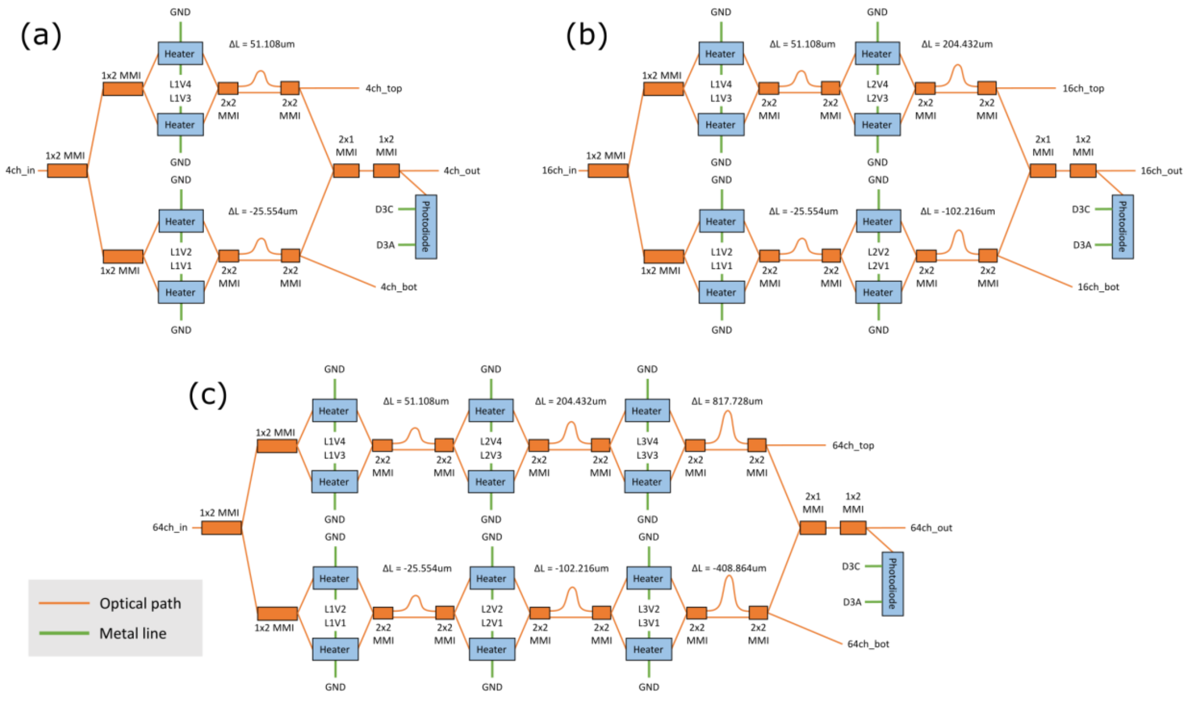

Supplementary Figure 3. Schematic of dFT spectrometers. Block diagram of the fabricated 1-stage (a), 2-stage (b), and 3-stage (c) dFT spectrometers fabricated all on a single chip. Orange denotes optical paths and passive optical components, while blue denotes active photonic components (heaters and photodetectors), and green denotes electrical wiring (metal traces).

spectrometers (Supplementary Figure 2a and 2b), and the same procedure as above was used to determine their insertion losses. The corresponding loss of the 2-stage dFT spectrometer was 6.8 ± 1.2 dB and the 1-stage dFT spectrometer was 5.5 ± 0.8 dB. The loss per stage was determined via linear regression to be 1.7 dB/stage, with a standard deviation error on this parameter of 0.4 dB (extracted from the covariance).

We note that the final 2 × 2 MMI's on the top and bottom arms incur a 3 dB loss penalty on the total insertion loss of the spectrometer. In future designs, it is possible to place an additional optical switch on both top and bottom arms to guide 100% of the light to the beam-combiner (this switch-state would depend on whether the input of the 2 × 2 MMI comes from the top or bottom port) to solve this issue.

Supplementary Note 3: Modulation efficiency estimates for thermo-optic and electro-optic phase shifters

For thermo-optic modulators, we assume a constant temperature rise of 60°C in the Si waveguide (with $dn/dT = 1.8 \times 10^{-4}$ K^{-1}) induced by local heating elements and an operating wavelength of 1550 nm. From these figures, a modulation efficiency (induced phase per unit waveguide length) is 433 rad/cm.

For electro-optic modulators[3], we assume a typical $V_\pi L_\pi = 1$ V·cm and applied voltage of 100 V. From this, the resulting modulation efficiency is 314 rad/cm.

To estimate the modulation efficiency of direct waveguide path modulation, we have a group index of n_g = 4.25 for the SOI waveguide geometry we employed, which yields a modulation efficiency of 172,281, a factor of 398 and 548 times greater than that of the thermo-optic and electro-optic shifters, respectively.

Supplementary Note 4: Temperature dependence of the dFT spectrometer

By detuning the thermoelectric heating/cooling element below the dFT chip, we performed a set of measurements and spectral reconstructions of a single-frequency laser at 1560.0nm. Using matrices A_1 and A_2 both measured at T = 25.00 °C and the temperature detuned interferogram measurements y_1 and y_2 (temperatures ranging from 25.00 °C to 23.87 °C), we used the elastic-D_1 method to reconstruct the spectrum.

For sparse (narrowband) inputs, the temperature detuning produced only a shift in the center wavelength of the single frequency laser, and otherwise did not produce significant spectral noise. Using this data, we characterized the first-order temperature sensitivity to be -85.2 ± 1.3 pm/°C, as shown in Supplementary Figure 4.

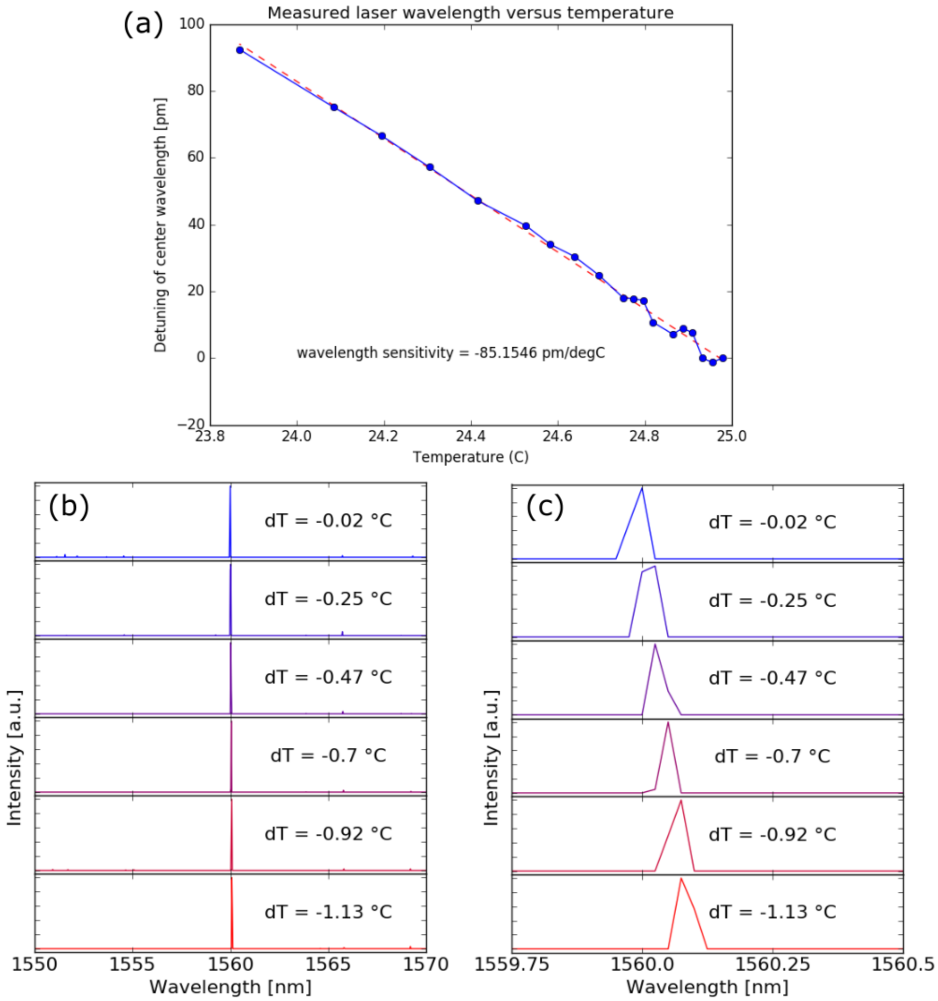

Supplementary Figure 4. Temperature dependence of narrowband dFT reconstructions. (a) measured detuning of the center wavelength for different temperatures as measured by a thermistor near the photonic chip. Images at bottom show the reconstructed laser line for a number of different temperatures across the full 20 nm band (b) and a zoomed in image is provided to show the change in center wavelength (c).

Supplementary Note 5: Insertion loss estimates from Supplementary Table 1

5-stage dFT: The insertion loss value was estimated from the measured loss per switching stage (1.7 ± 0.4 dB per stage) and the insertion loss of the 64-channel device (9.1 ± 1.7 dB).

32-channel MZI array[4] (Velasco, *et al.*): Insertion loss was estimated from the reported 4 dB/cm propagation loss, average waveguide length of 0.565 cm, and equal power splitting amongst 32 channels. This is a lower bound, as additional losses from power-splitting elements (1 × 2 MMI's) and waveguides leading up to the spirals are not included.

Spectrometer type	Spectral channel count	Spectral resolution	Bandwidth	Insertion loss per channel*	Power consumption	On-chip detector integration
3-stage dFT (*this work*)	64	24.6 GHz	2.47 THz	9.1 ± 1.7 dB	99 mW	Y
5-stage dFT (*projected*)	1024	1.5 GHz	2.47 THz	12.4 ± 2.6 dB	165 mW	Y
32-channel MZI array with fixed OPL[4]	32	5.0 GHz	0.094 THz	>17.3 dB	N/A	N
32-channel MZI array with fixed OPL[5]	32	2.3 GHz	0.035 THz	> 22.6 dB	N/A	N
MZI array with fixed OPL[6]	42	57.9 GHz	0.54 THz	> 32.9 dB	N/A	N
Thermo-optic FTIR[7]	18[†]	380 GHz	7 THz	6.7 dB	2.5 W	N
Arrayed waveguide grating[8]	50	25 GHz	1.26 THz	34 dB	N/A	N
Arrayed waveguide grating[9]	6	99.1 GHz	0.801 THz	11.8 dB	N/A	Y
Arrayed waveguide grating[10]	6	202 GHz	1.2 THz	9.8 dB	N/A	Y
Arrayed waveguide grating[12]	8	400 GHz	3.2 THz	10.8 dB	N/A	N
Planar concave grating[9]	16	397.5 GHz	9.3 THz	18.0 dB	N/A	Y
Random spectrometer[11]	25	100 GHz	3.28 THz	> 14 dB	N/A	N

Supplementary Table 1. Comparison of on-chip spectrometers. Side-by-side comparison of the demonstrated 64-channel dFT spectrometer along with previously reported MZI array FTIR, thermo-optic FTIR, arrayed waveguide grating, and random spectrometers. *Values in the "Insertion loss per channel" column account for the power loss per channel in dispersive spectrometers[8,9,11] when measuring broadband inputs and the loss from power-splitting in MZI array Fourier transform spectrometers[4–6,13]. [†]For Fourier transform spectrometers with continuously tunable arms, we define the effective spectral channel count as the bandwidth divided by the minimum spectral resolution. Plus-minus symbols denote one standard deviation errors across the entire 1550-1570 nm band.

32-channel MZI array[5] (Herrero-Bermello, *et al.*): Insertion loss was estimated from the reported 4 dB/cm propagation loss, average waveguide length of 1.89 cm, and equal power splitting amongst 32 channels. This is a lower bound, as additional losses from power-splitting elements (1 × 2 MMI's) and waveguides leading up to the spirals are not included.

42-channel MZI array[6] (Nedeljkovic, *et al.*): Insertion loss was estimated from the reported 8.8 dB/cm propagation loss, approximate waveguide length of 1.9 cm (estimated from reported 0.95 cm^2 device footprint), and equal power splitting amongst 42 channels. This is a lower bound, as additional losses from power-splitting elements (1 × 2 MMI's) and bending losses are not included.

Thermo-optic FTIR[7] **(Souza, *et al.*):** Insertion loss estimated from the reported 2 dB/cm propagation loss, 30.407 mm arm length of the thermally tuned interferometer, and 0.3 dB loss per y-junction (2 total).

50-channel AWG[8] **(Cheben, *et al.*):** Insertion loss calculated from the reported 17 dB insertion loss and the power splitting amongst 50 channels.

6-channel AWG[9] **(Ryckeboer, *et al.*):** Insertion loss calculated from the reported 4 dB insertion loss and the power splitting amongst 6 channels.

6-channel AWG[10] **(Muneeb, *et al.*):** Insertion loss calculated from the reported average 2.0 dB insertion loss and power splitting amongst 6 channels.

8-channel AWG (Bogaerts, *et al.*): Insertion loss calculated from averaging the minimum (center channel) insertion loss (1.1 dB) and maximum (outer channel) insertion loss (2.4 dB) and power splitting amongst 8 channels.

16-channel planar concave grating[9] **(Ryckeboer, *et al*.)**: Insertion loss calculated from the average reported 6 dB insertion loss and the power splitting amongst 16 channels.

Random spectrometer[11] **(Redding, *et al.*):** Since no values for the insertion loss were reported, we simply display the loss due to power splitting (14 dB) and note that the true insertion loss is larger than this value.

Supplementary References

1. Shalev-Shwartz, S. & Ben-David, S. *Understanding Machine Learning: From Theory to Algorithms*. (Cambridge University Press, 2014).
2. Andersen, M., Dahl, J. & Vandenberghe, L. CVXOPT: Python Software for Convex Optimization. (2016).
3. Ding, R. *et al.* Demonstration of a low $V_{\pi}L$ modulator with GHz bandwidth based on electro-optic polymer-clad silicon slot waveguides. *Opt. Express* **18,** 15618–15623 (2010).
4. Velasco, A. V *et al.* High-resolution Fourier-transform spectrometer chip with microphotonic silicon spiral waveguides. *Opt. Lett.* **38,** 706–708 (2013).
5. Herrero-Bermello, A. *et al.* Temperature dependence mitigation in stationary Fourier-transform on-chip spectrometers. *Opt. Lett.* **42,** 2239–2242 (2017).
6. Nedeljkovic, M. *et al.* Mid-infrared silicon-on-insulator Fourier-transform spectrometer chip. *IEEE Photonics Technol. Lett.* **28,** 528–531 (2016).
7. Souza, M. C. M. M., Grieco, A., Frateschi, N. C. & Fainman, Y. Fourier transform spectrometer on silicon with thermo-optic non-linearity and dispersion correction. *Nat. Commun.* **9,** 1–8 (2018).
8. Cheben, P. *et al.* A high-resolution silicon-on-insulator arrayed waveguide grating microspectrometer with sub-micrometer aperture waveguides. *Opt. Express* **15,** 2299–2306 (2007).
9. Ryckeboer, E. *et al.* Silicon-on-insulator spectrometers with integrated GaInAsSb photodiodes for wide-band spectroscopy from 1510 to 2300 nm. *Opt. Express* **21,** 6101–6108 (2013).
10. Muneeb, M. *et al.* III-V-on-silicon integrated micro - spectrometer for the 3 μm wavelength range. *Opt. Express* **24,** 9465–9472 (2016).
11. Redding, B., Liew, S. F., Sarma, R. & Cao, H. Compact spectrometer based on a

disordered photonic chip. *Nat. Photonics* **7,** 746–751 (2013).
12. Bogaerts, W. *et al.* Silicon-on-insulator spectral filters fabricated with CMOS technology. *IEEE J. Sel. Top. Quantum Electron.* **16,** 33–44 (2010).
13. Podmore, H. *et al.* Demonstration of a compressive-sensing Fourier-transform on-chip spectrometer. *Opt. Lett.* **42,** 1440–1443 (2017).
